# Is the Antibacterial Activity of Multi-Walled Carbon Nanotubes (MWCNTs) Related to Antibiotic Resistance? An Assessment in Clinical Isolates

**DOI:** 10.3390/ijerph18179310

**Published:** 2021-09-03

**Authors:** Pasqualina Laganà, Giuseppa Visalli, Alessio Facciolà, Marianna Pruiti Ciarello, Antonio Laganà, Daniela Iannazzo, Angela Di Pietro

**Affiliations:** 1 Department of Biomedical and Dental Sciences and Morphofunctional Imaging, University of Messina, 98125 Messina, Italy; gvisalli@unime.it (G.V.); afacciola@unime.it (A.F.); marianna.pruiti@gmail.com (M.P.C.); anto94.al@gmail.com (A.L.); adipietr@unime.it (A.D.P.); 2 Department of Electronic Engineering, Industrial Chemistry and Engineering, University of Messina, 98125 Messina, Italy; diannazzo@unime.it

**Keywords:** multi-walled carbon nanotubes (MWCNTs), antimicrobial properties, antibiotic resistance

## Abstract

Antimicrobial resistance has spread globally, compromising the treatment of common infections. This feature is particularly harmful for nosocomial pathogens that can survive on hospital surfaces. Research studies have been conducted to evaluate new materials that are able to counteract the microbial growth and the colonization of the hospital environment. In this context, nanotechnologies have showed encouraging applications. We investigated the antibacterial activity of multi-walled carbon nanotubes (MWCNTs), both pristine (p) and functionalized (f), at concentrations of 50 and 100 μg mL^−1^, against bacterial strains isolated from hospital-acquired infections, and this activity was correlated with the antibiotic susceptibility of the strains. The inhibiting effect of MWCNTs occurred for both types and doses tested. Moreover, f-MWCNTs exerted a greater inhibiting effect, with growth decreases greater than 10% at 24 h and 20% at 48 h compared to p-MWCNTs. Moreover, a lower inhibitory effect of MWCNTs, which was more lasting in Gram-positives resistant to cell wall antibiotics, or temporary in Gram-negatives resistant to nucleic acid and protein synthesis inhibitors, was observed, highlighting the strong relation between antibiotic resistance and MWCNT effect. In conclusion, an antimicrobial activity was observed especially for f-MWCNTs that could therefore be loaded with bioactive antimicrobial molecules. However, this potential application of CNTs presupposes the absence of toxicity and therefore total safety for patients.

## 1. Introduction

Antimicrobial resistance represents one of the most important current public health challenges worldwide. Many bacterial strains have developed resistance to most antibiotics used in therapy, and antibiotic-resistant strains have spread throughout the world. New resistance mechanisms are emerging and spreading globally, compromising the treatment of common infectious diseases. A certain number of infections, such as pneumonia, tuberculosis and sepsis, are becoming harder or even impossible to treat due to the lowered efficacy of antibiotics [1]. As highlighted by the World Health Organization (WHO), this issue represents a health emergency in both high- and low-income countries, affecting anyone of any age, and in a 2020 report, the WHO’s new Global Antimicrobial Surveillance System (GLASS) revealed the widespread occurrence of antibiotic resistance [1]. The Center for Disease Control and Prevention (CDC) affirms that, in 2019, in the USA, at least 2.8 million people are infected with antibiotic-resistant bacteria or fungi, and more than 35,000 people die as a result [2]. This aspect is particularly important for nosocomial pathogens, causing consistent increases of mortality, hospitalization times and costs for health systems [3]. A large number of studies have highlighted that hospital surfaces (beds, beside tables, carts, door handles, taps, mobile phones, computers, humidifiers for oxygen therapy, etc.) can host many nosocomial pathogens and could play an important role in the onset of hospital-acquired infections [4,5,6,7,8,9,10].

This worrying issue has induced researchers to evaluate new materials and strategies for successfully contrasting microbial growth and the colonization of the hospital environment. In this context, nanotechnologies have showed encouraging applications. Previous studies have focused attention on various nanosized antibacterial agents, such as metal and metal oxide nanoparticles (silver and silver oxide, titanium dioxide, zinc oxide, gold, calcium oxide, silica, copper oxide and magnesium oxide), that are able to inactivate microorganisms [11,12,13,14,15,16,17]. In addition, many studies have highlighted the high antimicrobial activity of carbon-based nanoparticles. In particular, early studies have indicated that fullerenes, single-walled carbon nanotubes (SWCNTs) and graphene oxide (GO) nanoparticles exhibit potent antimicrobial properties influenced by the size and surface area of these nanomaterials [18,19,20].

Carbon nanotubes (CNTs) are compounds of almost pure carbon that have been used for many purposes due to their different chemical and physical characteristics. They are used as sensing devices, energy production and storage systems, and, in the medical field, they are used as potential drug-delivery vehicles, especially in cancer therapy [21,22,23,24]. Moreover, interesting antimicrobial properties are associated with CNTs that have attracted relevant attention [25,26,27,28,29].

Some studies have identified oxidative stress (producing bacterial membrane damage) as one of the possible causes of the antimicrobial properties of CNTs [30,31,32,33,34]. However, recent studies have shown that the mechanical interaction of carbon-based nanomaterials with bacteria, and not oxidative stress, is the main antimicrobial activity of these compounds [35,36,37,38,39]. In particular, the first evidence of the strong antimicrobial activity of CNTs was obtained by treatment of *Escherichia coli* with SWCNTs, which caused severe membrane damage and bacterial death [40]. In a subsequent study, the same author highlighted the importance of the size of CNTs in this activity. Both SWCNTs single- and multi-walled CNTs (SWCNTs and MWCNTs) were studied as antibacterial agents against *E. coli*, and the results showed that SWCNTs exerted greater antibacterial action than MWCNTs. According to the authors, owing to their smaller diameter, SWCNTs could better penetrate into the cell wall than MWCNTs. Moreover, they highlighted that the differences between the two types of CNTs could also be attributable to the greater surface area, per unit of mass, of the SWCNTs [19].

For all of these reasons, CNTs are promising antibacterial candidates for a wide range of hospital applications. For example, surface-immobilized CNTs could be used to produce linens, furnishings and, above all, new composite materials for medical devices, hindering the colonization of pathogens.

The purpose of this study was to investigate the antibacterial activity of homemade MWCNTs (both pristine and functionalized) against 27 bacterial strains, isolated from hospital-acquired infections (HAIs), characterized by a different degree of antibiotic resistance, namely totally drug resistant (TDR), extensively drug resistant (XDR) and multidrug resistant (MDR). This is in order to more realistically evaluate their potential use in the fight against HAIs, considering the wide circulation of multidrug-resistant bacteria in the hospital environment.

## 2. Materials and Methods

### 2.1. Pristine and Functionalized MWCNTs

The biological effects of MWCNTs are strongly linked to their physicochemical properties, which in turn are related to post-synthetic modifications. In addition to homemade pristine MWCNTs (pMWCNTs), we also examined functionalized MWCNTs (fMWCNTs), such as MWCNT-COOH. The pMWCNTs were synthesized by catalytic chemical vapour deposition (CCVD), using Fe/Al_2_O_3_ as the catalyst and subsequently purified as reported previously [41,42]. Unlike our previous studies, oxidized carbon nanotubes (fMWCNTs) were prepared by using a mixture of sulfuric acid and nitric acid (1:3 vol ratio) for the treatment of purified pMWCNTs. This method increased the number of functional groups, allowing for wider water dispersion [43]. The inorganic fraction of MWCNTs (3.5–4%) was assessed by oxidative thermogravimetric analysis and was substantially represented by Fe_2_O_3_. The iron content was not bioavailable, as shown by abiotic and in vitro experiments [44].

The pMWCNTs had an average length of 10–20 μm and a diameter close to 15–30 nm, whereas the fMWCNTs were much shorter (average length between 200 and 1000 nm) and showed an external layer eroded at many points due to the oxidative insertion of terminal functional groups [41].

### 2.2. Experimental Conditions

The bactericidal capacity of both MWCNT types was assessed on strains of our collection that were isolated over time from clinical samples of patients admitted to the University Hospital (AOU) ‘G. Martino’ of the city of Messina. In particular, the study examined 27 different bacterial strains, namely 12 Gram-positive and 15 Gram-negative (Table 1). All strains were identified by commercially miniaturized biochemical tests (API 20 E, NE and API STAPH, bioMérieux, Marcy l’Etoile, France).

Based on preliminary tests to assess the bactericidal capacity of MWCNTs, 50 and 100 μg mL^−1^ concentrations were used. The work suspensions, prepared in peptone water, were obtained from stock suspensions (10 mg mL^−1^ in phosphate-buffered saline (PBS) at pH 7.2), preliminarily sterilized by autoclaving. In order to reduce MWCNT aggregation, due to the absence of repulsive electrostatic charges on the surface, the stock suspensions were submitted to sonication in an ultrasonic bath (Branson 3210) for 20 min (frequency 40 kHz), keeping the temperature low by using an ice bath. Just before experiments, the work suspensions in peptone water (2X) were further sonicated for 3 min, and the homogeneous suspensions were immediately dispensed into 96-well plates (50 µL/well).

For each bacterial strain, we set up 18–24 h broth cultures from which suspensions adjusted to a 0.5 McFarland turbidity standard (bioMérieux; corresponding to 1.5 × 10^8^ CFU mL^−1^) were prepared and added to the 96-well plates (50 µL for each preloaded MWCNT well). In each experiment, bacterial suspensions in peptone water without MWCNTs were included to assess bacterial growth in the basal condition, and suspensions of MWCNTs in the absence of bacterial suspensions were prepared as a negative control in order to test the sterility of the nanomaterials.

In the plates that were set up, the optical density (OD) at 550 nm was immediately recorded by using a Multiskan GO Microplate Spectrophotometer (Thermo Scientific) [45]. Then, the plates were incubated at 37 °C, and bacterial growth was assessed after 24 and 48 h.

After subtracting the zero-time OD in order to eliminate the interference caused by the nanotubes, the difference in absorbance was calculated for both 24 and 48 h to obtain bacterial growth in the basal condition (positive control) and in the presence of MWCNTs. All experiments were conducted at least in duplicate, and average values were used to assess bactericidal capacity of MWCNTs.

### 2.3. Antibiotic Susceptibility

The bacterial isolates were screened for antibiotic susceptibility, using the Kirby–Bauer test [46], performed with three replicates per isolate. The inoculum, consisting of 1.5 × 10^8^ CFU mL^−1^, was streaked over the surface of a Müeller–Hinton (MH) agar plate, using a cotton swab, and commercially available antibacterial disks (Oxoid) were used. The data in Supplementary Materials Table S1 report the assayed antibiotics, grouped in classes according to their mechanism of action [47].

After 48 h of incubation at 37 °C, the diameters of inhibition were measured with a precision calliper (Mitutoyo, Andover, UK) and averaged [48].

Overall, 24 broad-spectrum antibiotics were tested on all strains, while the following were used exclusively on the *S. aureus* strains: the cell wall inhibiting antibiotics amoxicillin (AML), oxacillin (OX) and penicillin (P); the protein synthesis inhibitors clindamycin (DA), erythromycin (E), lincomycin (MY), linezolid (LNZ), minocycline (MN), doxycycline (DXT) and methicillin (MET); and the semi-synthetic glycopeptide antibiotics teicoplanin (TEC) and vancomycin.

The Gram-negative strains were tested for the cell wall inhibiting antibiotics aztreonam (AZM), cefoxitin (FOX) and ceftazidime (CAZ); the disrupting membrane antibiotic colistin sulfate (CS); the nucleic acid inhibiting antibiotics nalidixic acid (NA), pipemidic acid (PI) and sulfamethoxazole + trimethoprim (SXT); and the protein synthesis inhibitors amikacina (AK), netilmicin (NET) and tobramycin (TOB).

Each bacterial species was classified as resistant (R), intermediate (I) or sensitive (S) according to the breakpoints established by the European Committee on Antimicrobial Susceptibility Testing (2021) [49]. For cinoxacin and sisomicin molecules, the breakpoints established by the Clinical Laboratory Standards Institute (2020) were used [50].

## 3. Results

**Scheme 1:**
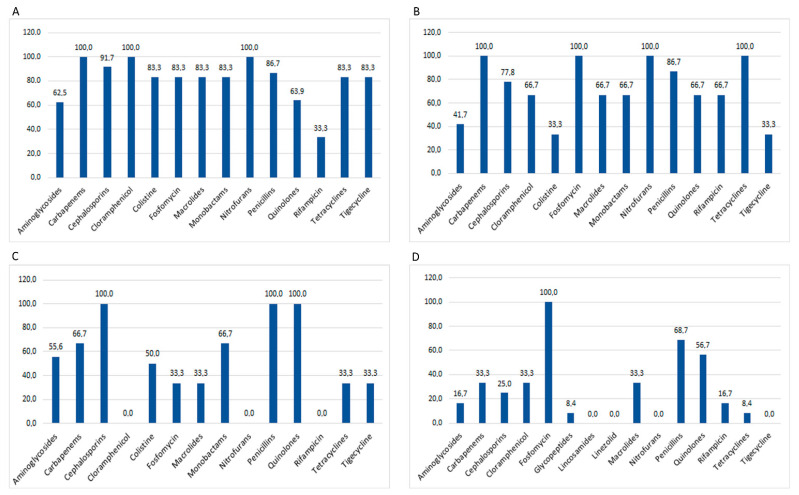
Mean percentages of antibiotic resistance to the tested strains. (**A**). *P. aeruginosa*; (**B**). *K. Pneumoniae*; (**C**). *E. coli*; (**D**). *S. aureus*.

The antibiotic susceptibility of the tested strains is shown in Scheme 1.

**Figure 1:**
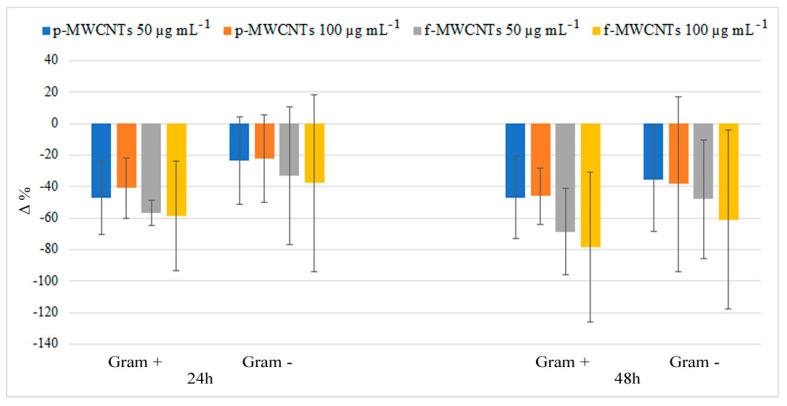
Values of the average percentage change (Δ%) after treatment at 24 and 48 h against the strains, divided into Gram-positive and Gram-negative bacteria.

The growth inhibiting effect occurred for both p- and f-MWCNTs and for both doses tested. Figure 1 reports the values of the average percentage change (Δ%) after treatment at 24 and 48 h against the strains, divided into Gram-positive and Gram-negative bacteria.

On all the tested strains, the f-MWCNTs exerted a greater inhibiting effect, with growth decreases averaging greater than 10% at 24 h and 20% at 48 h in comparison to pMWCNTs.

The antibacterial effect of MWCNTs was, on average, greater in the Gram-positive bacteria, for which, in comparison to the basal condition, the OD values in MWCNT-exposed suspensions were virtually halved at 24 h. The increase in inhibition as a function of dose was very small, and it was observed only for the most dispersible f-MWCNTs. Similar to what was observed for dose, the correlation between exposure time and antibacterial effect was weak in the Gram-positive bacteria, whereas, in the less sensitive Gram-negative bacteria, the observed percentage change showed, on average, an additional growth inhibiting effect of more than 50%, after exposure for 48 h.

However, the percentage change showed high inter-strain variability, as underlined by the coefficient of variation (CV). Even in the Gram-positive bacteria, consisting exclusively of *S. aureus* strains, the CV on average was equal to 50% after MWCNT exposure for 24 h. In particular, Δ% values in these strains ranged between −28.51 and −127.97 after exposure to fMWCNTs for 24 h.

As expected, MWCNT-induced inhibitory effects showed a more marked variability (CV on average greater than 100) in the Gram-negative bacteria, which included four different species. This higher inter-strain variability was also maintained by considering the three species separately, for which a greater number of strains had been examined. In particular, the CV was 127.1 in *P. aeruginosa* strains, which showed Δ% values ranging between −0.60 and −206.6 after exposure to fMWCNTs for 24 h. A similar variability was shown by *K. pneumoniae* whereas the CV in *K. oxytoca* strains was comparable to that observed in *S. aureus* strains.

To evaluate if the observed variability in growth inhibition was influenced by the antibiotic resistance of the different bacterial strains, preliminarily we compared the growth inhibiting effect of MWCNTs with the R/S ratio of each strain. Regardless of the mechanism of action of each drug, the R/S ratio was calculated by dividing the number of molecules to which the strain was resistant (R) or intermediate (I) with those to which it was sensitive (S).

The tested bacteria were previously isolated from clinical specimens in a hospital setting; the strains were multidrug resistant; and, in almost all of them, the R/S ratio was >1. Moreover, only in five strains did the R/S ratio highlight a higher number of molecules towards which the strain was sensitive compared to those towards which it was resistant. These included three strains of *S. aureus* (R/S ratio = 0.71), one *K. oxytoca* strain (R/S ratio = 0.53) and one *P. mirabilis* strain (R/S ratio = 0.69).

All *S. aureus* strains were resistant to amoxicillin, ampicillin and fosfomycin and sensitive to linezolid, nitrofurantoin and vancomycin, but the ratio of resistance/sensitivity against the other antibiotics was extremely variable.

With regard to the Gram-negative bacteria, all of the examined strains were resistant to ampicillin, amoxicillin + clavulanic acid and cefoxitin. On average, compared to those towards which they were sensitive, the number of antibiotics towards which they were resistant was three times higher. In the *P. aeruginosa* and *K. pneumoniae* strains, antibiotic resistance was highly represented. In particular, one strain of *K. pneumoniae* showed resistance to all the tested molecules (TDR), and one was sensitive only to the protein synthesis inhibitors amikacin and tigecycline (XDR), while two strains of *P. aeruginosa* were XDR. On average R/S values were 11.0, 17.0 and 2.4 in *P. aeruginosa*, *K. pneumoniae* and *K. oxytoca*, respectively.

**Figure 2:**
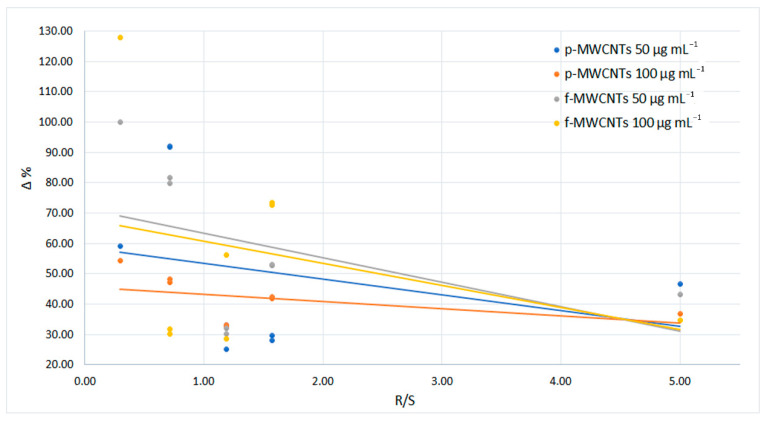
Correlation between the percentage increase of MWCNT antimicrobial activity and the R/S ratio of the studied bacterial strains.

The assessment of the inhibitor effect MWCNT-induced as a function of the antibiotic resistance of each *S. aureus* strain highlighted a close inverse correlation between the two variables, and a decreased effect was observed for higher R/S ratios. Significant Pearson’s correlation coefficients were observed for both doses of f-MWCNTs and the highest dose of p-MWCNTs (*p* < 0.05) and at both exposure times. As expected, the significance was maintained by using as independent variables both the number of molecules towards which the strain was resistant and the number towards which it was sensitive, highlighting an inverse and direct correlation, respectively. Moreover, as shown in Figure 2, a weaker effect of MWCNTs was observed with an increasing number of molecules towards which the strain was resistant, whereas the slopes of the regression lines confirmed the greater effectiveness of functionalized nanotubes. However, on analyzing the different classes of antibiotics separately, the growth inhibiting effect of MWCNTs was inversely related to drug resistance exclusively for molecules that act on the bacterial wall (r = 0.618; *p* = 0.03).

In Gram-negative strains, the antibacterial effect of the MWCNTs was not related to the R/S ratio calculated for all the tested molecules. However, the MWCNT-induced inhibitor effect in the strains exposed for 24 h was inversely related to drug resistance for both nucleic acid inhibitors (r = −0.460 and −0.492 for p- and f-MWCNT respectively; *p* < 0.05) and protein synthesis inhibitors (r = −0.510 and −0.530 for p- and f-MWCNTs, respectively; *p* < 0.01). However, unlike what was observed in the Gram-positive strains, the lower growth inhibitor effect of MWNCTs as a function of resistance to these two classes of antibiotics was temporary. After exposure for 48 h, no differences were observed as a function of drug resistance for both drug classes, as highlighted by the MWCNT-induced inhibiting effect being more marked in the drug-resistant strains. This rebound effect was confirmed by analyzing only the *P. aeruginosa* and *K. pneumoniae* strains, characterized by the highest R/S values. In these bacteria, the inhibitor effect of prolonged exposure to MWCNTs (48 h) was directly related to the R/S values, showing an increased susceptibility to engineered nanoparticles for the most drug-resistant strains (r = 0.551, *p* = 0.03 and r = 0.623, *p* = 0.01 in p- and f-MWCNTs, respectively).

Unfortunately, the small number of *K. oxytoca* and *P. mirabilis* strains did not allow us to evaluate the relationship between the MWCNT-induced effect and drug resistance for these Gram-negative strains with lower antibiotic resistance.

## 4. Discussion

To our knowledge, this is the first report to assess the MWCNT-induced antibacterial effect as a function of antibiotic resistance, a feature of fundamental importance when evaluating the potential use of nanomaterials for the production of devices to use in healthcare. Even if our homemade MWCNTs would have a less inhibiting effect compared to what observed by others authors, we evaluated the effects in presence of proteins which could neutralize the assayed antibacterial effect. Moreover, the relatively low inhibiting effects were due to the high prevalence of multidrug-resistant strains that, as demonstrated by our results, are inversely related to the antibacterial properties of MWCNTs.

To date, the spread of drug resistance in both clinical and environmental samples is a problem of primary importance in public health due to misuse of antibiotics in humans and animals and the horizontal intra-species and inter-species transmission of genes encoding drug resistance factors. As is well-known, acquired bacterial resistance involves several diverse mechanisms, including structural and biochemical changes. Referring to more specific reviews [51], we briefly report that, in these resistant phenotypes, due to chromosomal (vertical transfer) and extrachromosomal (horizontal plasmid transfer by conjugation) genes, different molecular pathways are involved in antibiotic resistance. These mechanisms are (i) production of enzymes responsible for drug degradation, (ii) change in membrane permeability that reduces the entry of water-soluble drugs by under-expression of porins, (iii) drug efflux determined by pump activation and, finally, (iv) reduced drug affinity consequent to a change in the bacterial target [52].

In addition to extended spectrum β-lactamases (ESBLs), often associated with factors responsible for resistance to aminoglycosides and trimethoprim/sulfamethoxazole), in several strains of Gram-negative bacteria, drug resistance is caused by a deficiency of porins due to transcriptional downregulation or mutation of the oprD gene. The mechanism slows down the uptake of nutrients and allows bacterial survival, decreasing the entry of hydrophilic antibiotics, such as β-lactams, aminoglycosides, tetracyclines and some fluoroquinolones [53,54,55,56].

Moreover, decreased intracellular drug concentration can be realized via export through membrane-located efflux pumps, and drug extrusion is achieved with several efflux pumps (ATP-binding cassette, resistance-nodulation-division, etc.) [57].

Finally, the MecA gene, which encodes for resistant proteins (penicillin-binding protein 2a), is present in several *S. aureus* strains (MRSA) that are able to continuously perform the transpeptidation required in peptidoglycan crosslinking for correct cell wall synthesis in the presence of antibiotics [58].

Previous studies have shown that the antimicrobial properties of CNTs depend particularly on their intrinsic structural characteristics. Indeed, it has been reported that a significant role is played by the surface charge, which is capable of interrupting cell membrane integrity [59]. Moreover, Bing et al. [60] found that positive and negative surface charges in carbon dots had an antibacterial activity that determined bacterial death, whereas Dwyer et al. [61] showed that the major factor responsible for inhibition of bacterial growth was the production of reactive oxygen species (ROS), such as hydroxyl radicals after interaction between charged dots and bacterial cells. In addition, Yang et al. [35] highlighted the role of CNT length on antimicrobial activity, confirming that the longer the SWCNTs, the better their aggregation and the stronger their antimicrobial properties. Finally, it is also plausible that the possible presence of impurities (especially metals used as catalysts in synthetic processes) [44] could play a certain role in the antimicrobial properties of CNTs.

Although all of these intrinsic characteristics affect the antimicrobial properties of CNTs, the above-described biochemical pathways regulating structural and biochemical changes in drug-resistant bacteria could be responsible for the lower inhibitory effect of MWCNTs, which is long-lasting in Gram-positive strains resistant to cell wall and disruptive membrane antibiotics or temporary in Gram-negative strains resistant to nucleic acid and protein synthesis inhibitors.

Therefore, even if it is beyond the aim of our study, it is plausible to believe that the inhibiting effect of the studied CNTs is not due to simple mechanical damage, as assumed by Yang et al. [35]. This effect due to MWCNT–bacteria interactions would determine the same outcome, regardless of antibiotic resistance. In response to exposure to stressors (i.e., drugs), antibiotic-resistant phenotypes could also express a greater ability to counteract more common stressors such as redox imbalance due to oxidative stress. As reported above, many authors [30,31,32,33,34] believe that the antimicrobial effect of CNTs is due to oxidative stress. In previous studies, the same MWCNTs assayed here were able to cause strong ROS overproduction in human cells, resulting in oxidative damages [42,62], and further study should aim to verify this hypothesis in bacterial cells.

Regardless of the variability of MWCNT-induced inhibition, our results highlight the greater efficacy of fMWCNTs, confirming how the greater water solubility resulting in greater dispersibility due to the presence of surface groups favors bacteria–CNT interactions. Furthermore, it is useful to underline that our homemade MWCNTs were subjected to functionalization by means of strong oxidation, remarkably increasing their bioreactivity due to surface defects in the hexagon ring of graphene caused by disruption of sp2 hybridization of the carbon atoms [44].

Although, as reported by Kang [19], the antibacterial effect of MWCNTs is weaker compared to SWCNTs at the same dose, these carbon-based nanomaterials have potential use in the production of composite materials to hinder microbial colonization.

Moreover, the presence of functional groups, in addition to allowing anchoring to the substrate (e.g., fabrics, plastics, paints, resins, etc.), allows conjugation to various molecules with disinfectant activity, enhancing the antimicrobial effect.

## 5. Conclusions

On overall, the results highlighted the potential use of functionalized MWCNTs in the production of composite materials to hinder microbial colonization. In any case, it is necessary to underline that the antibacterial effect is not exclusively attributable to the intrinsic characteristics of the MWCNTs (length, surface charge etc.) but also to the characteristics of the individual bacterial strains, such as structural and biochemical changes in drug-resistant bacteria. Referring to more specific reviews that only considered the potential use of carbon-based nanomaterials as carriers for drug delivery [21,22,23,24], MWCNTs can be loaded with bioactive antimicrobial molecules by means of functional groups. However, this potential application of CNTs presupposes the absence of toxicity against eukaryotic cells and thus total safety for patients, also considering the inter-individual variability of susceptibility [63]. To date, the results of CNT toxicity in humans and animals are controversial, and many studies have shown the capacity of carbon-based nanomaterials to induce oxidative stress in several human cells, such as alveolar and bronchial cells [41,64], neurons [42,65,66], etc. Taking this into consideration, it is necessary to make sure that the CNTs used to counteract microbial colonization are not released from the surfaces on which they are adsorbed or from the composite materials in which they are present.

## Figures and Tables

**Table 1:** Bacterial strains used in the experiment.

Gram-Positive Strains (*n*)	Gram-Negative Strains (*n*)
*Staphylococcus aureus* (12)	*Pseudomonas aeruginosa* (7) *Klebsiella pneumoniae* (3) *Klebsiella oxytoca* (4) *Proteus mirabilis* (1)

## Supplementary Materials

The following are available online at https://www.mdpi.com/article/10.3390/ijerph18179310/s1, Table S1: List of assayed antibiotics with the doses for each drug, grouped in classes according to their mechanism of action.
