# Supplementary material for: Is the Antibacterial Activity of Multi-Walled Carbon Nanotubes (MWCNTs) Related to Antibiotic Resistance? An Assessment in Clinical Isolates
Source: Int J Environ Res Public Health. 2021 Sep 3;18(17):9310. doi: 10.3390/ijerph18179310 (PMC8431017; doi:10.3390/ijerph18179310)
Supplement: Supplementary file 1 [file ijerph-18-09310-s001.zip › ijerph-1298130-supplementary.pdf]

International Journal of
Environmental Research and Public Health

MDPI

Supplementary Material for "Is the antibacterial activity of Multi-Walled Carbon Nanotubes (MWCNTs) related to antibiotic resistance? An assessment in clinical isolates." by Laganà P et al.

List of assayed antibiotics with the doses for each drug, grouped in classes according to their mechanism of action.

CELL WALL INHIBITING AND DISRUPTING MEMBRANE ANTIBIOTICS	**B-LACTAMS**	**PENICILLINS**	*Natural penicillins*	*penicillin (P, 1 unit,)*
			Aminopenicillins	*amoxicillin (AML, 10 μg)*
				ampicillin (AMP, 10 μg)
			Carboxipenicillins	*carbenicillin (CAR, 100 μg)*
			Ureidopenicillins	*mezlocillin (MEZ, 75 μg)*
				piperacillin (PRL, 100 μg)
			Penicillinase-resistant penicillins	*oxacillin (OX, 1 μg)*
				meticillin (MET, 5μg)
			Combinated penicillins	*amoxicillin + clavulanic acid (AMC, 30 μg)*
		**CEPHALOSPORINS**	*1st generation*	*cefazolin (KZ, 30 μg)*
			2nd generation	*cefoxitin (FOX, 30 μg)*
				cefuroxime (CXM, 30 μg)
			3rd generation	*cefotaxime (CTX, 30 μg)*
				ceftazidime (CAZ, 30 μg)
				ceftriaxone (CRO, 30 μg)
		**CARBAPENEMS**	*imipenem* (IMI, 10 μg)	
		**MONOBACTAMS**	*aztreonam* (AZM, *30 μg)*	
	**GLYCO - PEPTIDES**	*vancomycin (VAN, 30 μg)*		
		teicoplanin (TEC), 30 μg)		
	fosfomycin (FOS, 50 μg)			
	**POLYMIXINS**	*colistin sulphate* (CS, 10 μg)		
NUCLEIC ACIDS INHIBITING ANTIBIOTICS	**INHIBITING DNA TOPOISOMERASIS ANTIBIOTICS**	**QUINOLONES**	*cinoxacin (CIN, 100 μg)*	
			nalidixic acid (NA, 30 μg)	
			pipemidic acid (PI, 20 μg)	
		**FLUORQUINOLONES**	*ciprofloxacin (CIP, 5 μg)*	
			levofloxacin (LEV, 5 μg)	
			norfloxacin (NOR, 10 μg)	
			ofloxacin (OFX, 5 μg)	
	**INHIBITING FOLIC ACID**	**SULFONAMIDES**	*sulphamethoxazole + trimethoprim*	

	**SYNTHESIS ANTIBIOTICS**		(SXT, 25 µg)
	**INHIBITING RNA SYNTHESIS ANTIBIOTICS**	**RIFAMYCINS**	*rifampicin* (RD, 30 µg)
	**DNA INHIBITORS ANTIBIOTICS**	**NITROFURANS**	*nitrofurantoin* (F, 300 µg)
**PROTEIN SYNTHESIS INHIBITING ANTIBIOTICS**	**30S SUBUNIT INHIBITORS**	**AMINOGLYCOSIDES**	*amikacin* (AK, 30 µg)
			gentamycin (CN, 10 µg)
			netilmicin (NET, 30 µg)
			sisomicin (SIS, 30 µg)
			tobramycin (TOB, 10 µg)
		**TETRACYCLINES**	*doxycyclin* (DO, 30 µg)
			tetracycline (TE, 30 µg)
			minocycline (MN, 30µg)
		**GLYCYLCYCLINES**	*tigecycline (TGC, 15 µg)*
	**50S SUBUNIT INHIBITORS**	**MACROLIDES**	*azithromycin* (AZM, 15 µg)
			eritromycin (E, 15 µg)
		**LINCOSAMIDES**	*lincomycin* (MY, 2 µg)
			clindamycin (DA,10 µg)
		**OXAZOLIDINONES**	*linezolid* (LNZ, 10 µg)
		**PHENOLIC DERIVATIVES**	*chloramphenicol* (C, 30 µg)
